# Inhibition of Nek2 by Small Molecules Affects Proteasome Activity

**DOI:** 10.1155/2014/273180

**Published:** 2014-09-17

**Authors:** Lingyao Meng, Kent Carpenter, Alexis Mollard, Hariprasad Vankayalapati, Steven L. Warner, Sunil Sharma, Guido Tricot, Fenghuang Zhan, David J. Bearss

**Affiliations:** ^1^Center for Investigational Therapeutics, Huntsman Cancer Institute, Salt Lake City, UT 84112, USA; ^2^Tolero Pharmaceuticals Inc., 2975 Executive Parkway, Suite 320, Lehi, UT 84043, USA; ^3^Division of Medical Oncology, University of Utah, Salt Lake City, UT 84312, USA; ^4^Division of Hematology, Oncology, and Blood and Marrow Transplantation, Department of Internal Medicine, University of Iowa, Iowa City, IA 52242, USA

## Abstract

*Background*. Nek2 is a serine/threonine kinase localized to the centrosome. It promotes cell cycle progression from G2 to M by inducing centrosome separation. Recent studies have shown that high Nek2 expression is correlated with drug resistance in multiple myeloma patients. *Materials and Methods*. To investigate the role of Nek2 in bortezomib resistance, we ectopically overexpressed Nek2 in several cancer cell lines, including multiple myeloma lines. Small-molecule inhibitors of Nek2 were discovered using an in-house library of compounds. We tested the inhibitors on proteasome and cell cycle activity in several cell lines. *Results*. Proteasome activity was elevated in Nek2-overexpressing cell lines. The Nek2 inhibitors inhibited proteasome activity in these cancer cell lines. Treatment with these inhibitors resulted in inhibition of proteasome-mediated degradation of several cell cycle regulators in HeLa cells, leaving them arrested in G2/M. Combining these Nek2 inhibitors with bortezomib increased the efficacy of bortezomib in decreasing proteasome activity *in vitro*. Treatment with these novel Nek2 inhibitors successfully mitigated drug resistance in bortezomib-resistant multiple myeloma. *Conclusion*. Nek2 plays a central role in proteasome-mediated cell cycle regulation and in conferring resistance to bortezomib in cancer cells. Taken together, our results introduce Nek2 as a therapeutic target in bortezomib-resistant multiple myeloma.

## 1. Introduction

Nek2 is a serine/threonine protein kinase, belonging to the Nek family of cell cycle regulators [1]. The first member of this family, NIMA, was originally identified as a mutant preventing* A. nidulans* cells from entering mitosis. Thus, “NIM” stands for “never in mitosis” [2]. The Nek family has 11 members (Nek1-11), and Nek2 is the one with the highest sequence identity compared to NIMA [1]. Modern biochemical and proteomic data has shown that Nek2 is a core component of the human centrosome, and similar findings have also been reported for homologues of Nek2 in* Drosophila*,* Xenopus*, and mouse [3–6]. There is substantial evidence that Nek2 plays a key role in centrosome separation and promotion of the cell cycle from G2 to M phase [7–10]. Because the ubiquitin-proteasome system has been previously targeted with the proteasome inhibitor bortezomib in breast cancer [11–13], a few groups began to study and have subsequently reported that Nek2 regulates cell cycle progression in breast cancer cell lines [14–16].

While the effectiveness of proteasome inhibition in breast cancer continues to be evaluated, bortezomib continues to be a mainstay treatment for relapsed refractory MM [17, 18]. In spite of bortezomib's usefulness in improving overall survival in some patients, as many as one-third of relapsed MM patients do not respond to bortezomib and those that do respond often develop resistance [18–20]. For this reason, we sought to identify those genes related to myeloma drug resistance and disease relapse in a previous report. Among the genes studied, we found that Nek2 most accurately predicted poor prognosis, cell proliferation, and drug resistance in* ex vivo* and* in vitro* models of multiple myeloma [21].

Although several groups have tried to validate Nek2 as a therapeutic target using both small molecules and siRNA, few of them actually achieved efficient inhibition of Nek2 by small molecules [16, 22–25]. In this study, we identify a series of potent and selective inhibitors of Nek2, derived from a kinase-focused library screening approach. This approach provided us with selective, orally available small molecule inhibitors of Nek2, including HCI-2184, HCI-2388, and HCI-2389. All three of the compounds are related and have a pyrimidine scaffold as their core pharmacophore. These compounds inhibited proteasome activity* in vitro* and mitigated bortezomib resistance induced by Nek2 overexpression. Taken together, the data suggest that Nek2 plays an important role in the uncontrolled proliferation of MM cells and introduces Nek2 as a therapeutic target in relapsed refractory MM cells resistant to bortezomib.

## 2. Materials and Methods

### 2.1. Generation of Stable Nek2 Overexpressing (OE) Cell Lines

The Nek2 coding sequence was purchased and subcloned from a pCMV6-Entry vector (OriGene). Restriction enzymes AsiSI and XhoI were used to ligate the* NEK2* gene into the pCMV6-GFP vector (OriGene). The correct sequence of pCMV6-NEK2-GFP was verified by sequencing. Plasmid was generated in Top 10 cells (Invitrogen) and the plasmid was purified using the Small Scale Plasmid DNA Purification Kit (QIAGEN). Purified pCMV6-NEK2-GFP was used to transfect HeLa cells in 6-well plates, using Lipofectamine 2000 (Invitrogen). We chose to transfect HeLa cells with the pCMV6-NEK2-GFP plasmid because a previous report indicated the successful transfection of plasmids into NT2/D1 and HeLa cells using Lipofectamine 2000 without visible toxicity [26]. The final concentration of plasmid was 0.4 *μ*g/mL and the cell density was 8 × 10^5^ cells per well. G418 (Invitrogen) was added to DMEM medium with final concentration of 1 mg/mL. HeLa cells were cultured in G418 containing medium for one month. Clones were then isolated and continuously cultured as stable Nek2 OE HeLa cells. The same process was conducted using the pCMV6-GFP vector to establish stable GFP OE HeLa cells.

Other Nek2 OE multiple myeloma cell lines, including ARP1, H929, and KMS28PE cells, were developed as described in our previous report [21]. As with the HeLa cells, the final concentration of plasmid was 0.4 *μ*g/mL and the cell density was 8 × 10^5^ cells per well. Three additional clones of the ARP-1 cell line, Nek2-OE, Nek2-knockdown (KD), and bortezomib-resistant lines were generated as described in our previous report [21].

### 2.2. Western Blot Analysis

Following the indicated treatments, cells were washed by cold 1 X PBS buffer and were lysed using NP-40 cell lysis buffer (Life Technology). Whole-cell lysates were prepared and subjected to Western blot analysis as described in our previous report [21]. Briefly, after incubation with primary antibodies (Cell Signaling), the blots were probed with HRP-secondary antibodies (abcam). The blots were then detected with an ECL Detection Kit (Amersham).

### 2.3. Proteasome Isolation and* In Vitro* Proteasome Activity Assays

The 26S proteasome was isolated from whole-cell lysates by ultracentrifugation as previously described [27]. Proteasome activity was tested either in 96-well plates or 384-well plates using the Proteasome-Glo Trypsin-Like Assay (Promega). The assay was performed according to the vendor's protocol, and the proteasome concentration was optimized to 0.25 *μ*g/mL.

### 2.4. *In Vitro* Nek2 Inhibition Assays

Compounds were incubated with human Nek2 kinase (Invitrogen) and then kinase activity was examined by the Kinase-Glo Luminescence Kinase Assay (Promega). The assay was performed according to the manufacturer's protocol in 384-well plates' format using 60 mM Nek2. Twelve different concentrations were set for each compound: 100 *μ*M, 30 *μ*M, 10 *μ*M, 3 *μ*M, 1 *μ*M, 300 nM, 100 nM, 30 nM, 10 nM, 3 nM, 1 nM, and 0.3 nM.

### 2.5. Cell Viability Assays

Cell viability was determined using the ATPlite 1Step Kit (PerkinElmer) in 96-well plates. The assay was performed according to the vendor's protocol. Cell viability was assessed by measuring live cell ATP activity.

### 2.6. Cell Cycle Analysis

Cell cycle analysis was performed as described [28]. HeLa cells were harvested and resuspended in Krishan's Buffer (0.1% sodium citrate, 50 *μ*g/mL propidium iodide, 20 *μ*g/mL RNase A, and 0.5% NP-40). Flow cytometry was conducted on a FACScan cytometer (Becton, Dickinson and Company). Collected data was analyzed by FlowJo 6.0b software (Tree Star, Inc.).

### 2.7. Statistical Analyses

Data was tested for statistical significance by unpaired *t*-tests using the Graph-Pad InStat Software. Data was considered statically significant when *P* < 0.05.

## 3. Results

### 3.1. Nek2 Overexpression Induced Bortezomib Resistance in HeLa Cells

**Figure 1:**
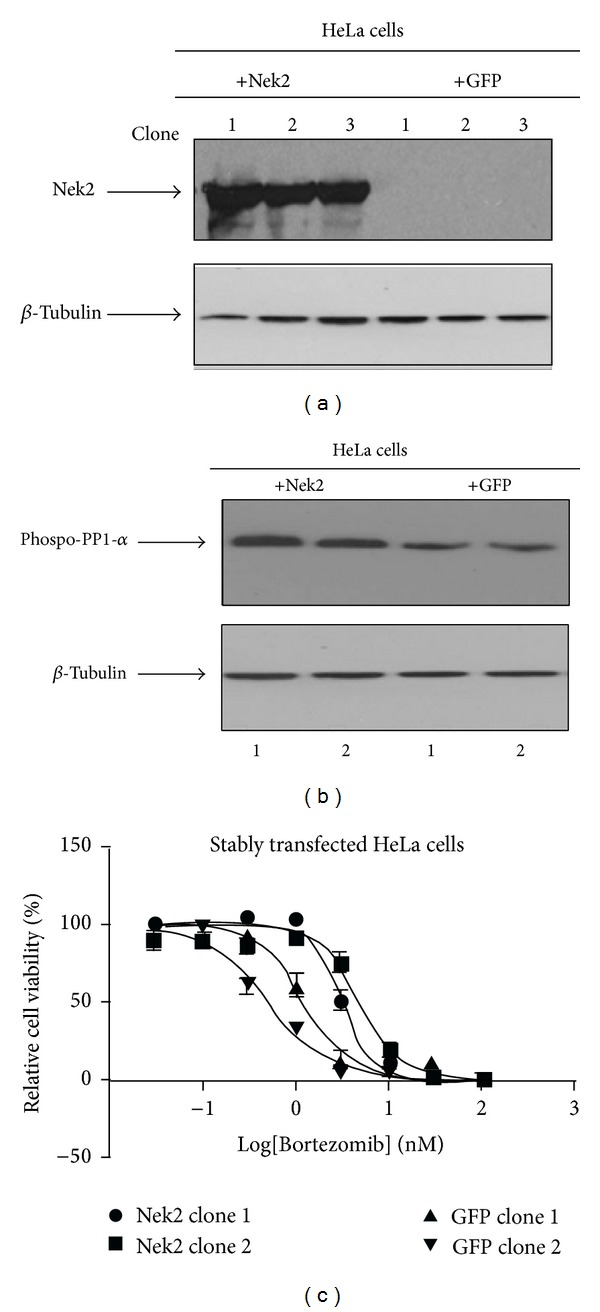
Nek2 overexpression causes HeLa cells to become resistant to bortezomib. (a) The* Nek2* gene was cloned into a GFP expression vector as described in Materials and Methods Section. HeLa cells were then transfected with either the Nek2-GFP plasmid or GFP expression vector alone. Anti-NEK2 antibody was used to confirm NEK2 overexpression as determined by Western blot. (b) Nek2 overexpression increased the level of phosphorylated PP1-α in the two surviving Nek2 transfected clones. (c) Nek2-GFP transfected HeLa cells were resistant to bortezomib treatment compared to GFP-transfected clones. Bortezomib was used to treat HeLa cells with the concentration range from 100 nM to 0.03 nM. Within this range, at any given concentration of bortezomib, Nek2-transfected clones yielded higher cell viability than GFP-transfected clones.

We previously reported that bortezomib resistance is accompanied with Nek2 upregulation in MM patients [21]. To confirm this correlation, we used the constructed Nek2-GFP plasmid to transfect HeLa cells, and Nek2 overexpression was first confirmed by Western blot (Figure 1(a)). The lower band in the blots corresponds to endogenous Nek2 whereas the larger band corresponds to the Nek2-GFP plasmid. Increased phosphorylation of PP1-*α*, a known substrate of Nek2 [29], was also verified by Western blot in Nek2-OE cells (Figure 1(b)).

The two most viable HeLa Nek2-OE clones and HeLa GFP-OE clones were selected for the following experiments. Bortezomib was used to treat these HeLa cells in a 96-well plate under different concentrations (100 nM, 30 nM, 10 nM, 3 nM, 1 nM, 0.3 nM, 0.1 nM, and 0.03 nM) with 0.1% DMSO as control. After 72 hours, cell viability was examined by the ATP lite assay. At every concentration of bortezomib, Nek2-OE clones yielded higher cell viability than GFP clones (Figure 1(c)). These data suggest that bortezomib resistance was induced by Nek2 overexpression in HeLa cells, which is consistent with our previously reported data [21].

### 3.2. Proteasome Activity Was Significantly Increased by Nek2 Overexpression

Because bortezomib is able to target cancer cells by proteasome inhibition [30], we hypothesized that Nek2 overexpression would increase proteasome activity in transfected cells and subsequently confer bortezomib resistance. To test this hypothesis, the 26S proteasome was isolated by ultracentrifugation from the stable Nek2-OE cells. Three different human MM cell lines, including ARP1, H929, and KMS28PE, were tested. Among them, we tested four verified clones of the ARP-1 cell line, including wild-type, Nek2-OE, Nek2-knockdown (KD), and bortezomib-resistant clones. These cell lines were generated and verified as described in our previous report [21].

**Figure 2:**
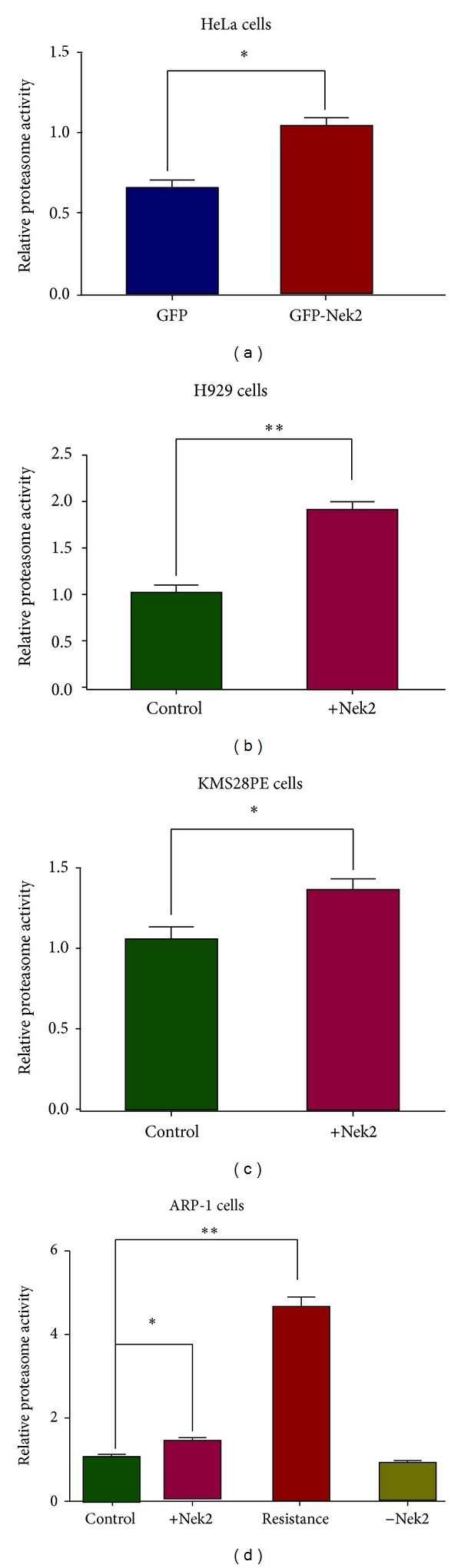
Nek2 overexpression elevates the proteasome activity in multiple cancer cell lines. (a) Proteasome activity is significantly increased in Nek2 overexpressed HeLa cells compared to GFP-transfected control. Proteasome activity was also significantly elevated in H929 (b), KMS28PE (c), and ARP-1 (d) cell lines compared to empty vector transfected (control). For the ARP-1 cell line, Nek-2-OE, NEK-2-KD, and bortezomib-resistant clones were tested in addition to wild-type cells. The 26S proteasome was isolated by ultracentrifugation and the proteasome activity was determined by Proteasome-Glo Assay. (d) For ARP1 cells, the bortezomib-resistant cells (third column in (d)) showed higher proteasome activity. For Figures 2(a)–2(d), *P < 0.05, **P < 0.01.

*In vitro* proteasome activity from the isolated proteasome was tested by the Proteasome-Glo Trypsin-Like Assay. For all the studied cell lines, the proteasome activity of the Nek2-OE cells was significantly higher than the control (GFP-treated for HeLa cells and empty vector treated cells for H929, KMS28PE, and ARP-1 cells). Bortezomib resistant ARP-1 cells exhibited the highest proteasome activity (Figures 2(a)–2(d)). These results support our hypothesis and imply that Nek2 overexpression is one of the mechanisms behind increased proteasome activity in bortezomib-resistant MM cell lines.

### 3.3. Nek2 Inhibitors Reduce the* In Vitro* Proteasome Activity in Nek2-Expressing Cell Lines

**Figure 3:**
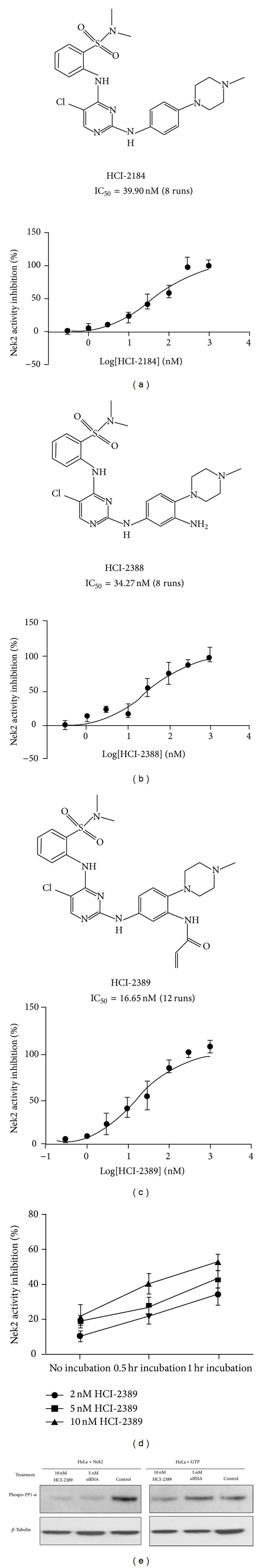
Novel Nek2 Inhibitors significantly Inhibit Nek2's activity. (a), (b), and (c), three compounds, HCI-2184, HCI-2388, and HCI-2389 were designed by virtual screening. Synthesized compounds were validated by NMR and MS. The abilities of the three compounds to inhibit Nek2 kinase were tested by Kinase-Glo Assay. (d) HCI-2389 acts as an irreversible Nek2 inhibitor. A 0.5 hr incubation of HCI-2389 and Nek2 kinase increased the ability of HCI-2389 to inhibit Nek2. This effect was more pronounced when HCI-2389 was incubated with Nek2 kinase for 1 hr. (e) 10 nM HCI-2389 treatment for 72 hours greatly decreased the level of phosphorylated PP1-α in both Nek2 overexpressed HeLa cells and GFP controls. The effect was equal to or greater than treatment with 5 nM Nek-2 siRNA.

A focused screening library of ~2000 compounds was assembled from an in-house collection of previously synthesized kinase inhibitors utilizing a single concentration screening approach in a Nek2 biochemical kinase assay. This yielded four compounds with Nek2 kinase inhibition greater than 80% at 10 *μ*M. These 4 hits were filtered by physical property calculations,* in vitro* ADME, and kinase selectivity filters to give one compound, HCI-2184, that was selected for further experiments examining the role of Nek2 in drug resistance. Using the Kinase-Glo assay, we determined the IC_50_ of HCI-2184 and found that it was <100 nM. Structure-based optimization was used to synthesize additional analogues of HCI-2184 and three compounds were selected as potential leads, HCI-2184, HCI-2388, and HCI-2389, all of which yield an average IC_50_ under 50 nM (Figures 3(a)–3(c)). Among them, HCI-2389 was the most potent Nek2 inhibitor. This is most likely due to its irreversible binding mode of action (see Supplementary Figure S1 available online at http://dx.doi.org/10.1155/2014/273180) which was tested by the Kinase-Glo assay with the drug preincubated with Nek2. The Nek2 inhibitory activity of HCI-2389 was significantly increased after preincubation times as short as 0.5 hours (Figure 3(d)).

Based on its potency, HCI-2389 was selected to treat the Nek2-OE HeLa cells. We performed Western blots to measure the downstream effects of Nek2 inhibition caused by HCI-2389 treatment. We found that the level of phosphorylated PP1-*α* was significantly decreased in HeLa cells treated with concentrations as low as 10 nM of HCI-2389 for 72 hours (Figure 3(e)).

**Figure 4:**
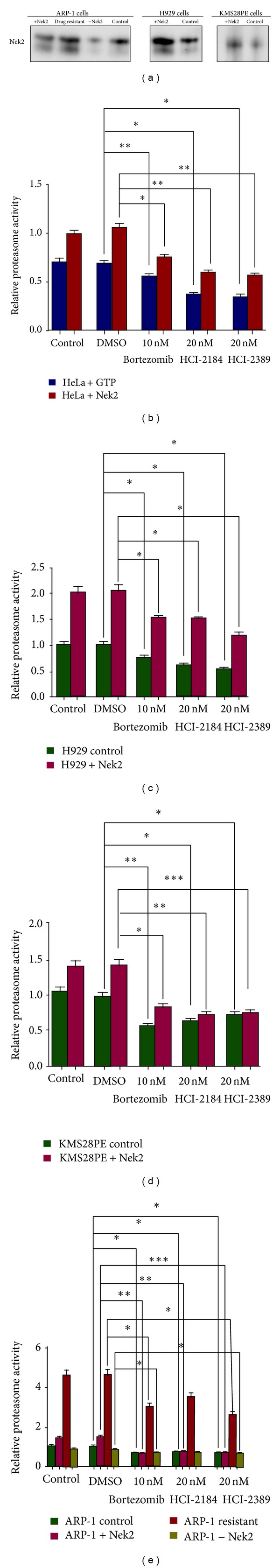
Novel Nek2 inhibitors effectively decrease the proteasome activity* in vitro* for multiple cancer cell lines. (a) Nek2 was found to be involved in the 26S proteasome in cancer cell lines. The 26S proteasome was isolated by ultracentrifugation and the presence of Nek2 in the 26S proteasome was determined by Western blot. (b), (c), (d), and (e), Incubation of HCI-2184 and HCI-2389 significantly inhibits the proteasome activity for HeLa cells (b), H929 cells (c), KMS28PE cells (d), and ARP1 cells (e). (e) The irreversible Nek2 inhibitor HCI-2389 worked better than HCI-2184 in decreasing the proteasome activity for ARP1 cells that are resistant to bortezomib treatment. For Figures 4(b)–4(e), *P < 0.05, **P < 0.001, ***P < 0.0001.

Our observation that Nek2 overexpression increased proteasome activity led us to ask whether our Nek2 inhibitors were able to inhibit this increased activity. We tested this hypothesis by isolating the 26S proteasome by ultracentrifugation from multiple Nek2-OE cells as described in Materials and Methods. Interestingly, Nek2 was found to be involved in the proteasome complex (Figure 4(a)), suggesting a possible direct interaction with the proteasome components. In accordance with this notion, the levels of Nek2 associated with the proteasome were proportional to overall Nek2 levels in cells (Figure 4(a)). It is important to note this relationship was not as clear in the KMS28PE cell line, where levels of both endogenous and transfected Nek2 were not as apparent (Figure 4(a)). To test the effect of our Nek2 inhibitors on proteasome activity, the compounds were incubated with the isolated proteasome followed by the Proteasome-Glo assay. Our Nek2 inhibitors inhibited proteasome activity* in vitro* at a level similar to bortezomib (Figures 4(b)–4(e)). Based on these results, we concluded that the Nek2 inhibitors were responsible for the decrease in proteasome activity in the Nek2-OE cancer cell lines tested.

**Figure 5:**
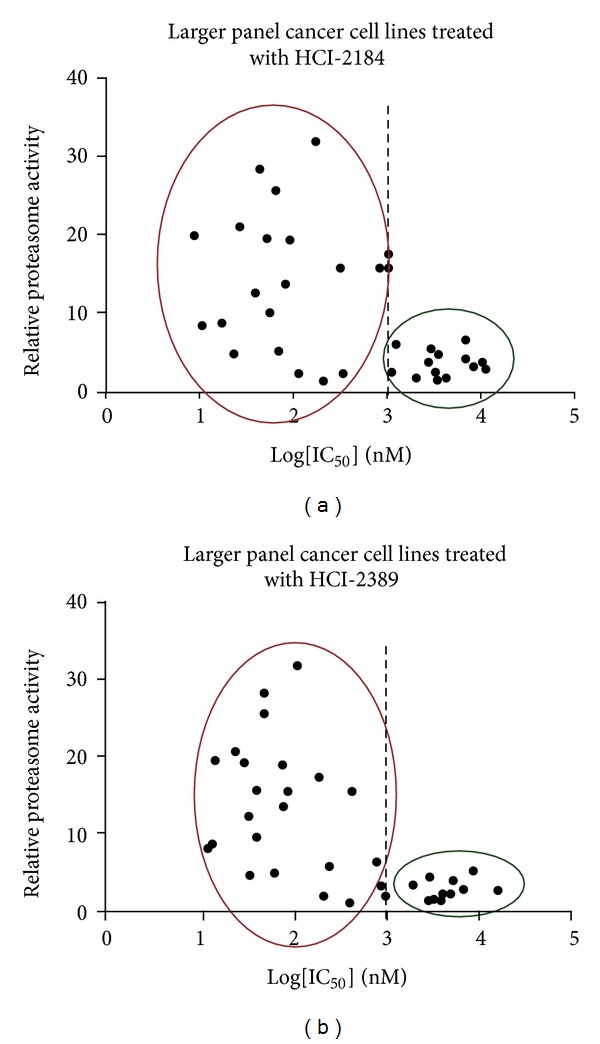
The sensitivity of cancer cell lines to Nek2 inhibitors is correlated with their proteasome activity. (a) Cell lines sensitive to HCI-2184 treatment had, on average, higher proteasome activity compared to resistant cell lines. (b) Cell lines sensitive to HCI-2389 treatment had, on average, higher proteasome activity compared to resistant cell lines. These cancer cell lines were selected from the 150 cell lines in our lab, based on whether or not they were sensitive to both HCI-2184 or HCI-2389. “Sensitive” was defined as an IC_50_ value of 1 *μ*M or lower. The 26S proteasomes were isolated by ultracentrifugation and proteasome activity measured by Proteasome-Glo Assay.

We further studied the effect of Nek2 inhibitors on additional cell lines using the ATP-lite cell viability assay. We treated a large panel of cell lines (*n* = 36) with our Nek2 inhibitors. These 36 cell lines were either responsive (“responsive” was defined as an IC_50_ value of less than 1 *μ*M) to both HCI-2184 and HCI-2389 or nonresponsive to either of the two inhibitors selected for the proteasome activity assay. Data analysis showed that although there was not a strict proportional relation between proteasome activity and Nek2 inhibitor responsiveness, the average proteasome activity of the sensitive cell lines was significantly higher than that of the nonsensitive cell lines (Figure 5).

### 3.4. The Combination of Bortezomib and Nek2 Inhibitors Reduces Proteasome Activity to a Greater Extent Than Either Drug Alone

**Figure 6:**
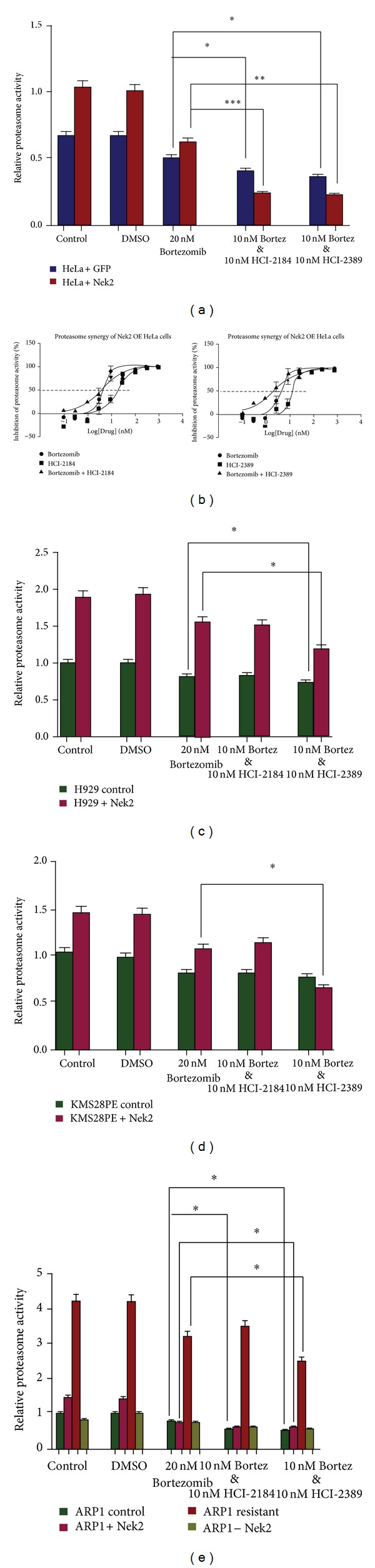
The combination of bortezomib and Nek2 inhibitors reduces proteasome activity to a greater extent than either drug alone. (a) The combination of bortezomib with either HCI-2184 or HCI-2389 significantly increased the effectiveness of bortezomib on Nek2-OE HeLa cells compared to GFP-transfected controls. (b) The combination of Nek2 inhibitors HCI-2184 or HCI-2389 and bortezomib inhibited proteasome activity in Nek2-OE HeLa cells to a greater extent than either drug alone. (c) and (d), the combination of Bortezomib and HCI-2389 decreased proteasome activity compared to untreated or DMSO treated H929 (c) or KMS28PE (d) cells treated with either empty vector (control) or Nek2 overexpressing (+Nek2) cells. (e), the combination of bortezomib and HCI-2389 decreased proteasome activity in empty vector (control), Nek2 plasmid (+Nek2), and Nek2 siRNA knockdown (−Nek2) ARP1 cells. The combination also resulted in a significant decrease in proteasome activity in ARP1 cells resistant to bortezomib (ARP1 resistant). For (a) and (c)–(e), *P < 0.05, **P < 0.001, ***P < 0.0001.

We next combined our Nek2 inhibitors with bortezomib in the proteasome-Glo assay to determine whether Nek2 inhibitors could be used in combination with bortezomib. In treated HeLa cells, both HCI-2184 and HCI-2389 significantly increased the effectiveness of bortezomib in inhibiting proteasome activity at concentrations as low as 10 nM (Figure 6(a)). Additionally, dose response studies confirmed that these two Nek2 inhibitors shift the inhibition curve of bortezomib (Figure 6(b)).

For the other three cell lines studied, including H929, KMS28PE, and ARP1, HCI-2389 was also able to increase the efficacy of bortezomib, while HCI-2184 had less of a synergistic effect (Figures 6(c)–6(e)). Again, the irreversible binding of HCI-2389 provides a possible explanation for this difference between compounds.

These results provide evidence that proteasome activity can be inhibited to a greater extent when combining Nek2 inhibitors with bortezomib, compared to bortezomib alone, suggesting that Nek2 is a potential molecular target that might be used in combination with bortezomib to treat MM patients.

### 3.5. Nek2 Inhibitors Prevented Mitotic Proteins from Being Degraded by Proteasome, Causing G_2_/M Phase Arrest

Many proteins are targeted and degraded by the proteasome for mitotic entry as well as mitotic exiting [31–34]. Degradation of Cyclin B and Cdc2 plays a significant role in mitotic regulation [35–38]. Previous research has shown that downregulation of proteasome activity lead to the accumulation of Cyclin B [39], triggered by the overexpression of Hec1, a substrate of Nek2. As Nek2 overexpression elevated proteasome activity and Nek2 inhibition decreased it, we set out to evaluate the levels of a few key mitotic regulators targeted by the proteasome.

**Figure 7:**
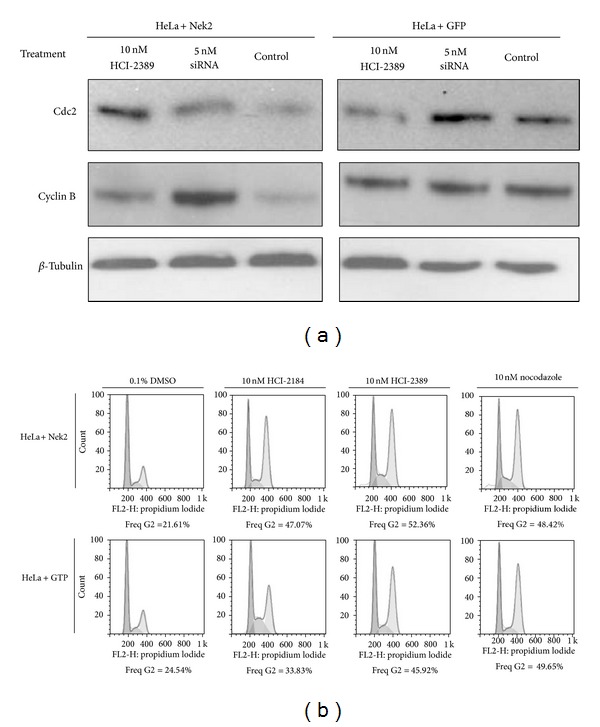
Novel Nek2 inhibitor prevents Cdc2 and Cyclin B from being degraded by the proteasome, catching HeLa cells in G2/M phase. (a) Western blot showed that the degradation of Cdc2 and Cyclin B was inhibited by treatment of 10 nM HCI-2389. This effect was significant in Nek2 overexpressed HeLa cells. (b) Flow cytometry data showed that, treated by 10 nM HCI-2184 or 10 nM HCI-2389, around 50% HeLa cells stayed at G2/M phase. 100 nM nocodazole was used as the positive control.

In this experiment, cells were synchronized in mitotic phase, followed by treatment of Nek2 inhibitors for 72 hours. The levels of Cyclin B and Cdc2 were then evaluated by Western blot. Both Cyclin B and Cdc2 were found to be downregulated by Nek2 overexpression (Figure 7(a)), which is consistent with the finding that Nek2 overexpression causes increased proteasome activity. Further, treating the cells with HCI-2389 or Nek2-siRNA successfully inhibited the degradation of Cyclin B and Cdc2. This effect was not as dramatic in the GFP controls, which expressed only basal levels of Nek2.

Cell cycle analysis was performed to examine the effect of Nek2 inhibition on the cell cycle. The stably transfected HeLa cells were treated with 10 nM of HCI-2184 and HCI-2389 for 24 hours and then analyzed by flow cytometry. We found that almost 50% of the Nek2 inhibitor-treated cells were arrested in G2/M phase (Figure 7(b)). As before, HCI-2184 did not work as well as HCI-2389 in arresting HeLa GFP OE cells, and this is probably because HCI-2389 is a more specific Nek2 inhibitor than HCI-2184. Further research will be needed to elucidate the detailed inhibitory mechanisms of these compounds.

In summary, Cyclin B and Cdc2 were downregulated by Nek2 overexpression and Nek2 inhibition reversed this effect. Nek2 inhibitors, through inhibition of proteasome activity, inhibited Cyclin B and Cdc2 from being degraded. This resulted in cell cycle arrest in G_2_/M phase in the Nek2-OE cells.

## 4. Discussion

Although progress in the treatment of MM has been made in the past decade [40, 41], myeloma remains largely incurable with current therapeutic strategies. Bortezomib is one of the most effective chemotherapies for MM, but drug resistance remains a crucial problem with bortezomib treatment [17, 19, 20]. Little is known about the molecular mechanisms involved in this resistance. In our previous report, we used gene expression profiling in a variety of MM cases and identified Nek2 as the most significant gene associated with early relapse [21]. Other reports have similarly shown that Nek2 overexpression induces chemotherapeutic resistance* in vitro* [15, 42]. It is clear that there is an urgent need for exploring the mechanism linking the Nek2 kinase to drug resistance and the development of novel Nek2 inhibitors. To our knowledge, this study represents the first link connecting Nek2's biological function of regulating proteasome activity as the mechanism of bortezomib resistance in multiple myeloma. It is also the first to establish highly effective Nek2 inhibitors that successfully inhibit proteasome activity in cancer cell lines.

The 26S proteasome complex is a core component of the ubiquitin-proteasome system (UPS) of protein degradation. Ubiquitination regulates multiple cell cycle aspects including checkpoints control and cell growth progression [18, 43, 44]. The 26S proteasome is essential for the rapid elimination of the cell cycle regulators and the transcription factors such as NF-*κ*B, whose fast degradation is important to the proper cell processes [44, 45]. Cdk1 and Cdk2 drive progression through each cell cycle phase and G2/M transition in particular [38, 46]. The activation of Cdks greatly depends on the availability of their cyclin partners, and cyclin levels are strongly regulated by the UPS [32]. In addition, the UPS has been shown to regulate the Cdk inhibitors such as Wee1 [37, 46]. Studies have shown that two complexes are involved in the UPS regulation of cell cycle: the anaphase-promoting complex or cyclosome (APC/C) and the Skp1/Cullin-1/F-box protein complex (SCF) [34, 47, 48]. These two complexes have different cellular functions and play crucial roles in different cell phases. APC/C regulates the degradation of mitotic cyclins, such as Cyclin B1, and consequently inhibits Cdk1, leading cells to mitotic exit [41, 42].

Previous reports suggested that Nek2 primarily played a role in regulating centrosome separation [7–10]. Overexpression of active Nek2 induces premature splitting of centrosomes, while silencing of Nek2 blocks spindle and chromosome segregation. As centrosome separation is crucial for mitotic entry, Nek2 was thought to participate in cell cycle control. However, compared to other mitotic kinases, Nek2's function is relatively subtle and, in our study, neither suppression nor silencing of Nek2 expression dramatically affected the cell cycle. This has been the major obstacle for studying Nek2's biological function. In this research, we explored Nek2's function and we confirmed the correlation of Nek2 overexpression and bortezomib resistance in HeLa cells. Bortezomib exerts its effects on cancer cells by inhibiting proteasome activity. Subsequently, we hypothesized that Nek2's role in bortezomib resistance was related to increasing proteasome activity. Using multiple cancer cell lines, we showed that overexpression of Nek2 significantly elevated proteasome activity. Specifically, we found higher proteasome activity in bortezomib resistant ARP1 cells. This elevated* in vitro* proteasome activity is inhibited by our Nek2 inhibitors HCI-2184 and HCI-2389 which rescue drug resistance of Nek2-OE HeLa cells. However, the mechanism of how Nek2 regulates proteasome activity is still unknown and needs further investigation.

Together with the Polo and Aurora kinase families, the NIMA-related protein kinases (Neks) have been called the third family of mitotic kinases [2]. Previous studies suggest that Nek family members influence cell cycle progression by regulating Cyclin B and Cdc2 [2, 49, 50]. Here, we discovered Nek2 overexpression down-regulated both Cyclin B and Cdc2 by increasing activity of the proteasome. This finding may provide more information for further study of Nek2's function in the cell cycle regulation.

By examining the proteasome activity of multiple cancer cell lines, we have identified Nek2 upregulation as a potential mechanism for bortezomib resistance related to proteasome activity elevation. However, for ARP1 cells, the proteasome activity of the bortezomib resistant clone was higher than that of the Nek2 OE clone (Figure 2(c)). Therefore, other mechanisms aside from Nek2 upregulation may be involved in proteasome activity elevation. Further effort is needed to elucidate other proteasome regulators as potential drug targets for MM therapeutics.

Although we have synthesized several potent Nek2 inhibitors with demonstrated activity (Figure 3), these inhibitors need better selectivity to advance them as potential clinical candidates (Supplementary Figure S2).

In summary, we have discovered that high levels of Nek2 expression are at least partly responsible for elevated proteasome activity and subsequent bortezomib resistance in human MM treatment. More excitingly, we have shown that Nek2 inhibition results in proteasome activity suppression and cell cycle arrest. This research provides important knowledge for future studies of Nek2's biological function and provides potential solutions for bortezomib resistance in MM therapy.

## 5. Clinical Practice Points

Although progress in the treatment of MM has been made in the past decade [40, 41], myeloma remains largely incurable with current therapeutic strategies. Bortezomib is one of the most effective chemotherapies for MM, but drug resistance remains a crucial problem with bortezomib treatment. Little is known about the molecular mechanisms involved in this resistance. In our previous report, we used gene expression profiling in a variety of MM cases and identified Nek2 as the most significant gene associated with early relapse. Other reports have similarly shown that Nek2 overexpression induces chemotherapeutic resistance* in vitro*. It is clear that there is an urgent need for exploring the mechanism linking the Nek2 kinase to drug resistance and the development of novel Nek2 inhibitors. To our knowledge, this study represents the first link connecting Nek2's biological function of regulating proteasome activity as the mechanism of bortezomib resistance in multiple myeloma. It is also the first to establish highly effective Nek2 inhibitors that successfully inhibit proteasome activity in cancer cell lines.

## Supplementary Material

The Supplementary Material provides further detail about the small molecule Nek2 inhibitors used in the studies. The first Supplementary Figure depicts the structural binding mode of HCI-2389 in the ATP-binding pocket of Nek2 and rationalizes the irreversible bond between the small molecule and Cys22 on Nek2. The second Supplementary Figure shows the selectivity of HCI-2184 and HCI-2389 against representative panel of 39 kinases. Percent inhibition is shown on the graph at a single screening concentration (200 nM).
