# Supplementary material for: Inhibition of Nek2 by Small Molecules Affects Proteasome Activity
Source: Biomed Res Int. 2014 Sep 17;2014:273180. doi: 10.1155/2014/273180 (PMC4182079; doi:10.1155/2014/273180)
Supplement: Supplementary file 1 — The Supplementary Material provides further detail about the small molecule Nek2 inhibitors used in the studies. The first Supplementary Figure depicts the structural binding mode of HCI-2389 in the ATP-binding pocket of Nek2 and rationalizes the irreversible bond between the small molecule and Cys22 on Nek2. The second Supplementary Figure shows the selectivity of HCI-2184 and HCI-2389 against representative panel of 39 kinases. Percent inhibition is shown on the graph at a single screening concentration (200 nM). [file 273180.f1.pdf]

Structural Model of HCI-2389 Binding to Nek2

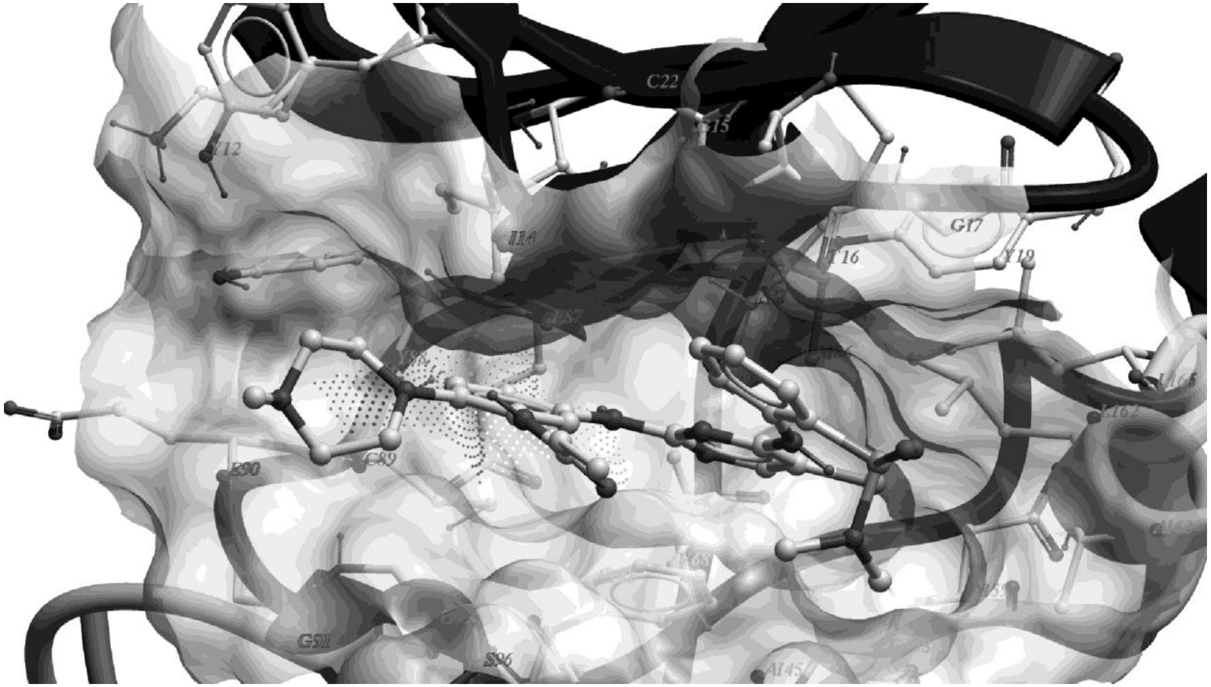

Supplementary Fig. S1. Binding Mode of HCI-2389.

The acryl amide functional group of HCI-2389 is positioned with in 5.6 Å distance from Cys89. Pyrimidine series having Michael acceptor substitution at the solvent binding site participate in covalent bonding with Cys22 presumably increasing the selectivity for Nek2 was confirmed from this structural and kinase profiling.

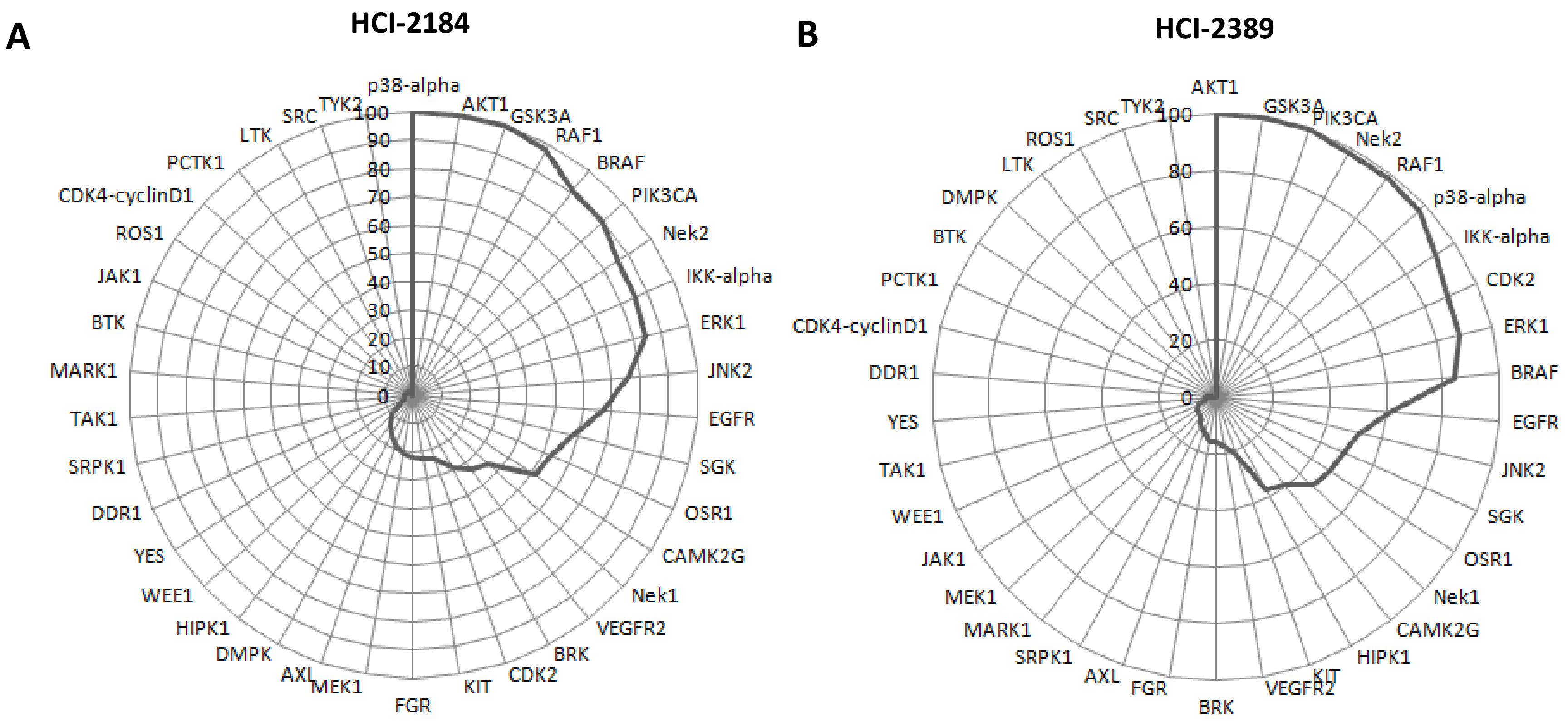

Supplementary Fig. S2. Selectivity of Nek2 inhibitors.

Four hundred fifty-one kinases were screened for their inhibition percentage when treated by HCI-2184 (A) or HCI-2389 (B) at 200nM. Thirty-nine representative (function in cancer) kinases were selected for mapping. HCI-2184 and HCI-2389 showed similar selectivity while HCI-2389 has more selectivity due its irreversible binding of action.
